# FLASH effect tissue sparing comparison for X-ray, hadron, and VHEE irradiation modalities

**DOI:** 10.1038/s41598-026-46934-8

**Published:** 2026-07-13

**Authors:** Atia Ibrahimi, Stefan Both, Marc-Jan van Goethem, Francesco Romano, Alexander Gerbershagen

**Affiliations:** 1 Particle Therapy Research Center (PARTREC), Department of Radiation Oncology, University Medical Center Groningen, University of Groningen, 9747 AA Groningen, The Netherlands; 2 Sezione di Catania, Instituto Nazionale di Fisica Nucleare, 95123 Catania, Italy; 3 John Adams Institute for Accelerator Science, Department of Physics, University of Oxford, Oxford, OX1 3RH UK

**Keywords:** FLASH radiotherapy, Ultra-high dose rates, Monte carlo simulations, Healthy tissue sparing, Irradiation modalities, Biophysics, Cancer, Medical research, Oncology, Physics

## Abstract

FLASH-RT is an irradiation modality using Ultra-High-Dose-Rates, where a healthy tissue sparing effect is observed. Different particle types have distinct characteristics in lateral and distal dose deposition, which can affect the amount of healthy tissue sparing by the FLASH effect. However, few comparative studies investigate this impact. In this Monte Carlo-based study, we compare different irradiation modalities in a simple geometrical model and demonstrate how dose, dose-rate, and particle type influence the volume of healthy tissue benefiting from FLASH. The results indicate that less conformal modalities, such as X-rays and VHEE, benefit more from the effect than hadrons. Despite this, FLASH X-rays and FLASH VHEE still deposit higher dose to healthy tissue than conventional hadrons. Assuming a FLASH-triggering dose threshold and dose-rate threshold from literature, we found that hadrons require a target dose of 20 Gy and target dose-rate of 75 Gy/s to achieve healthy tissue sparing, about twice as high as for VHEE and X-rays. The results imply that highly fractionated treatments cannot be applied under current assumptions of FLASH-triggering dose and dose-rate thresholds. Future studies using clinical dose distributions could explore the use of multiple angles, fractionation possibilities, and relative biological effectiveness (RBE) for more specific clinical cases.

## Introduction

External beam radiotherapy can be performed with different radiation beams, such as photons, electrons, protons, and carbon. Additionally, the use of helium beams is being increasingly investigated. Compared to protons, helium beams provide sharper penumbras, which allow for a better conformality. Additionally, helium has the advantage of producing less fragmentation than carbon^1^, which leads to a tail in the depth-dose distribution after the Bragg peak. The objective of these treatment modalities is to maximise damage to the tumour while minimising damage to surrounding healthy tissue.

FLASH-RT represents a novel approach to tumour treatment that employs external irradiation at ultra-high dose-rates (UHDR). This approach has been observed to result in a healthy tissue sparing effect, while it has been demonstrated that the tumour tissue response is maintained at these UHDRs^2^, which opens the therapeutic window. This phenomenon is known as the FLASH effect. The precise mechanism underlying this effect remains unclear and is currently under investigation. One hypothesis suggests that the impact of FLASH may vary depending on the protein class involved, potentially leading to distinct effects on normal and tumour tissue^3^. The time-averaged dose-rates for conventional radiotherapy are typically in the order of a few Gy/min, whereas for FLASH-RT these are 40 Gy/s or higher^2^.

A multitude of factors influence the determination of the dose-rate, which can be interpreted in different ways due to specific irradiation methods being employed. For instance, when considering the influence of time structure within a pulsed beam, which can be more relevant for electron UHDR beams accelerated by LINACs^4^. In some pencil beam scanning studies, the time-averaged dose-rate is selected as a means of defining the dose-rate, which is a modest method and typically the lowest of the dose-rate definitions^5^.

There are strong indications that a dose threshold and dose-rate threshold must be reached in order to trigger the FLASH effect. Studies often assume a dose threshold of about 10 Gy^6^. The dose-rate threshold is typically assumed to be 40 Gy/s, although other values, such as 100 Gy/s, can be found in literature as well^7^. Further research is needed to establish the precise definitions of the threshold values, which can be difficult as the FLASH effect depends on dose, dose-rate, and endpoint^8^.

There are few comparative studies investigating the effect of different particle types on the amount of tissue spared by the FLASH effect. The different modalities each have their own characteristics in terms of lateral and longitudinal dose distributions. The manner in which the particles deposit their dose can influence the FLASH effect, which defines the aim of the present study. The objective of this study is to show the influence dose deposition characteristics of different modalities have on the FLASH effect and to identify which modalities could benefit the most from the FLASH effect.

## Materials and methods

### Simulation setup

**Fig. 1:**
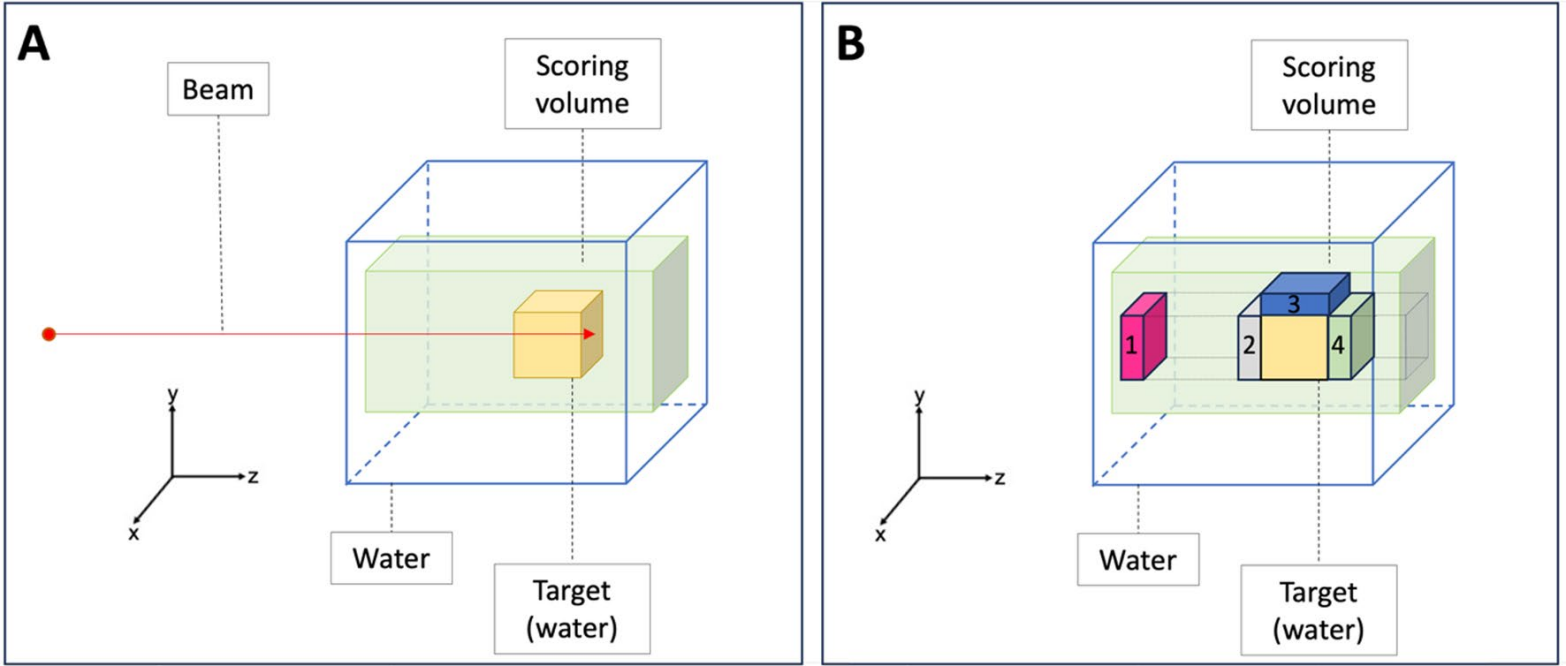
Schematic of simulation setup used for MC simulations (not to scale). (**A**) Beam in z-direction irradiating a 2 ⋅ 2 ⋅ 2 cm^3^ target volume (yellow) in a surrounding volume of water. Scoring volume indicated in green. (**B**) Four different regions of interest at: the entrance of the volume (1), anterior to the target (2), lateral to the target (3), and posterior to the target (4). Regions 1,2, and 4 are 2 ⋅ 2⋅ 1 cm^3^ (x⋅y⋅z), and ROI 3 is 2 ⋅ 1⋅ 2 cm^3^.

Monte Carlo simulations were conducted using Topas^9,10^, which is a Geant4^11–13^ -based tool. The simulation setup is illustrated in Fig. 1A and will be described in greater detail below. A 2 ⋅ 2 ⋅ 2 cm^3^ target volume of water is defined in a surrounding volume of water, with the centre of the target situated at a depth of 9 cm in the water. The target and the surrounding volume represent tumour tissue and healthy tissue respectively. A scoring volume is indicated in green and is divided into 121 ⋅ 121 ⋅ 360 voxels of 0.5 ⋅ 0.5 ⋅ 0.5 mm^3^ each, where the dose is scored by using the built-in DoseToMedium scorer. The range cuts in the simulation were set at 0.05 mm and the Geant4 reference physics list QGSP_BIC_HP was implemented.

### Simulation parameters

The target is irradiated by the beam originating from the left in Fig. 1A. The simulated particle sources provide the beams of protons, ^4^He^2+^, ^12^C^6+^, very high energy electrons (VHEE), and X-rays. In clinical settings, the utilisation of multiple irradiation angles is a common practice. In this study, a single irradiation angle is used for all modalities. The main reason to use a single angle is to simplify the comparison of the results. Moreover, the use of a single angle will allow to reach the threshold values that have been identified as necessary to trigger the FLASH effect. For the hadrons, multiple beam spots and energy layers were used to irradiate the target, whereas for VHEE, a single energy was employed. The X-ray field was shaped using a collimator. To cover the target in the longitudinal direction, 23 energy layers were used for the hadrons.

For the protons, the beam energies ranged from 102 MeV to 118 MeV. The beam energies for helium and carbon were 102–118 MeV/u and 194–223 MeV/u, respectively, such that their ranges matched those of the protons. The beam characteristics were derived from the proton beam at the University Medical Center Groningen (UMCG) Proton Therapy Center to mimic clinical conditions. Dependent on the energy, the standard deviation of the Gaussian energy spread ranged from 0.67% to 0.69%, and of the Gaussian spot size from 4.9 mm to 5.5 mm. For VHEE, a 150 MeV beam was used, with characteristics comparable to those of the hadrons. The X-ray spectrum ranged from 1.5 to 14 MeV, based on the 15 MV beam of the Elekta Synergy linac at the UMCG. The beam characteristics were chosen to cover the transverse dose profiles of the 2 ⋅ 2 cm^2^ field in the clinic.

The relative biological effectiveness (RBE) of all particle modalities, including protons, helium, and carbon ions, has not been considered. As an example, the spread-out Bragg peak (SOBP) of carbon ions is represented as a flat physical dose rather than a flat RBE-weighted dose. To achieve coverage in the transverse planes for both hadrons and VHEE, different spots were used. Nine spots were assumed, as in practice reaching FLASH dose-rates is easier with a minimal number of spots. The spots were arranged on a 3 ⋅ 3 grid centred on the target. The dose distribution of the central spot was obtained directly from the Monte Carlo simulation. For computational efficiency the remaining eight spots were generated in post-processing by translating this central-spot distribution to the corresponding grid positions. The energy layers and spots were exclusively focused on achieving maximum homogeneity within the target volume and not on steep penumbras. This made it possible that the centre of a spot lies (slightly) outside the target boundary, while the spot still overlaps the target. Allowing such spot placement increases achievable dose uniformity, compared to a distribution in which all spot centres remain strictly within the target. The trade-off is that such spot positioning outside of the target boundary can potentially increase the penumbras. With the use of one irradiation angle, conformality is best reached with the hadrons, as the application of different spot positions and energy layers facilitates uniform irradiation across the entire target. Conformality in the longitudinal direction will not be reached as effectively with VHEE and X-rays and the relatively high dose anterior to the target is unavoidable with VHEE and X-rays.

### Tissue sparing model

The tissue-sparing model employed in this study is based on a parameterisation by Böhlen et al.^14^, who derived FLASH modifying factors (FMFs) as a function of the dose applied from a review of the literature on in vivo FLASH experiments. Together with a threshold dose (*D*_*T*_), threshold dose-rate ($\:{\dot{D}}_{T}$), and an *FMF*^*min*^, which is the value to which the *FMF* converges at high doses, the *FMF* is given by Eq. (1).

1 $$\:{FMF\left(D,\dot{D}\right)}_{{FMF}^{min},\:{D}_{T},{\dot{D}}_{T}}=\left\{\begin{array}{c}1,\:\:D\le\:{D}_{T}\:or\:\dot{D}\le\:\:{\dot{D}}_{T}\\\:\left(1-{FMF}^{min}\right)\frac{{D}_{T}}{D}+{FMF}^{min},\:\:D>{D}_{T}\:and\:\dot{D}>\:{\dot{D}}_{T}\end{array}\right.$$

The dependence of *FMF* on dose-rate and dose-rate threshold is modelled using a step function. The *FMF* will be equal to one at *D*_*T*_ or lower, when the dose-rate $\:\dot{D}$ is equal to or lower than $\:{\dot{D}}_{T}$, or in the target (tumour tissue). If the dose *D* is greater than *D*_*T*_ and $\:\dot{D}$ greater than $\:{\dot{D}}_{T}$, the *FMF* will be less than one, converging to *FMF*^*min*^. The $\:{\dot{D}}_{T}$ utilised in this study is 40 Gy/s. Two sets of *D*_*T*_ and *FMF*^*mi*n^ values were used in this study, namely those for the mammalian without skin data set (*D*_*T*_ = 9.9 (± 0.9) Gy, *FMF*^*min*^ = 0.60 (± 0.05)) and the mammalian skin data set (*D*_*T*_ = 16.6 (± 1.3) Gy, *FMF*^*min*^ = 0.51 (± 0.03))^14^. In this study, it has been assumed that the FLASH effect is the same for different modalities, independently on the particle type, and will depend only on a dose threshold and dose-rate threshold. This implies that the *FMFs* are the same for the different particle beams at the same dose and dose-rate.

### Evaluation of FLASH effect

After retrieving the dose distributions in the scoring volume from the MC simulations, in post-processing the dose distributions were normalised in such a way that the minimum dose in the target did not fall below the limit of 1 Gy -3%.

In this study, the dose-rate is defined as the time-averaged dose-rate, i.e., the deposited dose divided by the total time required to deliver the entire irradiation, accounting for the energy layer switching time between the different spots. The normalised distribution allowed to vary target dose and target dose-rate, by applying a scaling factor, to achieve a target dose of 20 Gy and target dose-rate of 80 Gy/s. These values were chosen because they are well above the threshold values and the FLASH effect will be visible for all modalities, which also allows for the differences between the modalities to be shown. Selecting a dose and dose rate too close to the threshold would result in limited healthy tissue sparing due to FLASH, making it difficult to observe meaningful differences between modalities. The scaled dose and dose-rate are used for the determination of the *FMF*. In addition, some regions in the scoring volume described earlier were selected for further analysis. These regions of interest (ROIs) can be seen in Fig. 1B, which shows part of the schematic from Fig. 1A. The target is indicated in yellow, and the four numbered ROIs in pink, grey, blue, and green. ROIs 1,2, and 4 measured 2 ⋅ 2 ⋅ 1 cm^3^ (x ⋅ y ⋅ z), and ROI 3 measured 2 ⋅ 1 ⋅ 2 cm^3^. The statistical uncertainty within each ROI and the target, defined as the mean uncertainty of voxels receiving at least 20% of the maximum dose in that region, was below 2%.

The incorporation of healthy tissue sparing was achieved by utilising the *FMF* from Eq. (1), in conjunction with the *D*_*T*_ and *FMF*^*min*^ values. For the first 2 mm in the scoring volume, proximal to the target, the values from the mammalian skin data were used, as this represents the average human skin thickness^15^. For all other regions the values for mammalian without skin were used. An *FMF* value of 1 signifies that sparing will not occur, which will be observed in tumour tissue (given that the FLASH effect is a healthy tissue sparing effect) or in healthy tissue if the threshold conditions (dose or dose-rate) are not met. The resulting FLASH dose (*D*_*FLASH*_) is evaluated for each voxel as follows:

2 $$\:{{D}_{FLASH}}_{x,y,z}={FMF}_{x,y,z}\cdot\:\:{D}_{x,y,z}$$

In this context, *D* represents the dose in the absence of the FLASH effect.

Following scoring in the scoring volume, the processing of the dose distributions, and the inclusion of healthy tissue sparing factors, analyses have been performed within the scoring volume and ROIs. These analyses included overall dose distributions, dose-volume histograms (DVHs), and the effects of thresholds, target dose, and target dose-rate.

## Results

### Dose distributions

**Fig. 2:**
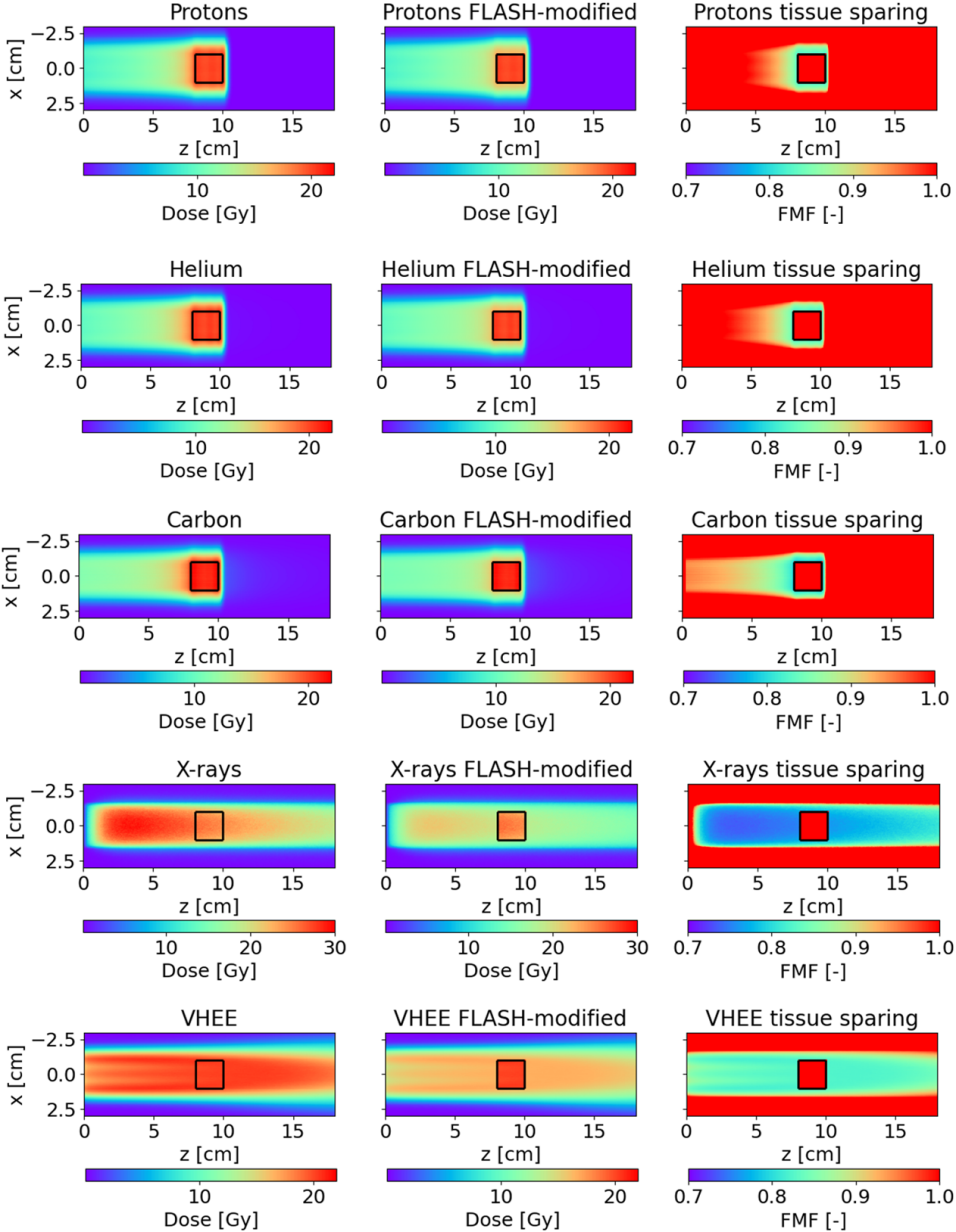
Dose distributions for all modalities for a target dose of 20 Gy at conventional dose-rate (left) and FLASH dose-rate (center) with the corresponding FMFs (right).

The output of the MC simulations after post-processing is presented in Fig. 2 for all modalities. The results are shown for a target dose of 20 Gy. The distributions in the left column assume a conventional dose-rate. The lateral penumbras (80 − 20%) vary from 0.41 cm to 0.84 cm between the modalities, with the sharpest penumbra for X-rays, and the largest penumbra for protons. The distal penumbras (80 − 20%) for the hadrons are 0.25 cm for protons and 0.26 cm for helium and carbon. In the same figure the *FMF*s are shown (see right column), where a target dose-rate of 80 Gy/s is assumed, together with the resulting FLASH-modified dose distributions (see centre column).

**Fig. 3:**
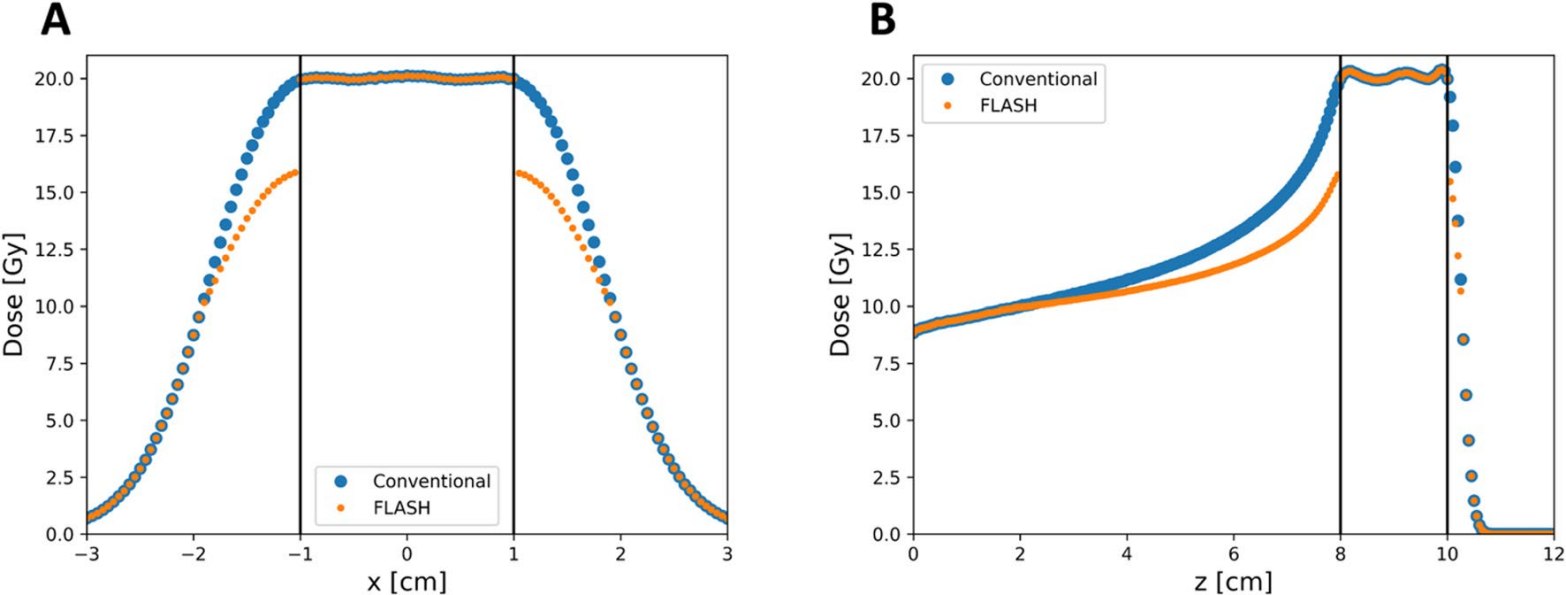
Dose distribution profiles for protons for a target dose of 20 Gy at conventional dose-rate and FLASH dose-rate of 80 Gy/s, with tumour region marked by black lines. The FLASH-modified dose distribution is more conformal, resulting in a lower dose to the healthy tissue. (**A**) Transverse (x-profile) at y = 0 cm and z = 9 cm. (**B**) Longitudinal (z-profile) at x = 0 cm and y = 0 cm.

Figure 3 shows the transverse profile at y = 0 cm and z = 9 cm and the longitudinal profile at x = 0 and y = 0 cm of the dose distribution for protons for both conventional and FLASH dose-rate, which demonstrates the sparing of healthy tissue.

### Dose and dose-rate requirements to see FLASH

Table 1 provides the minimum target dose (D_*min*_) and target dose-rate (DR_*min*_) required to observe the FLASH effect in any region of the healthy tissue and in ROI1 for all modalities, assuming a DR_*T*_ of 40 Gy/s.

**Table 1:** Minimum target dose (*D*_*min*_) and target dose-rate (*DR*_*min*_*)* required to see FLASH effect for different modalities, both for the entire healthy tissue volume and entrance ROI1, with *D*_*min*_ uncertainties propagated from the uncertainties in the FLASH model parameters.

Modality	Entire healthy tissue volume		ROI1	
	D_min_ [Gy]	DR_min_ [Gy/s]	D_min_ [Gy]	DR_min_ [Gy/s]
Protons	10 (± 1)	40	20 (+ 2 / − 1)	81
Helium	10 (± 1)	40	19 (+ 2 / − 1)	76
Carbon	10 (± 1)	38	17 (+ 1 / − 2)	67
X-rays	7 (+ 1)	27	10 (± 1)	38
VHEE	10 (− 1)	37	10 (± 1)	39

### Volume covered by FLASH

**Fig. 4:**
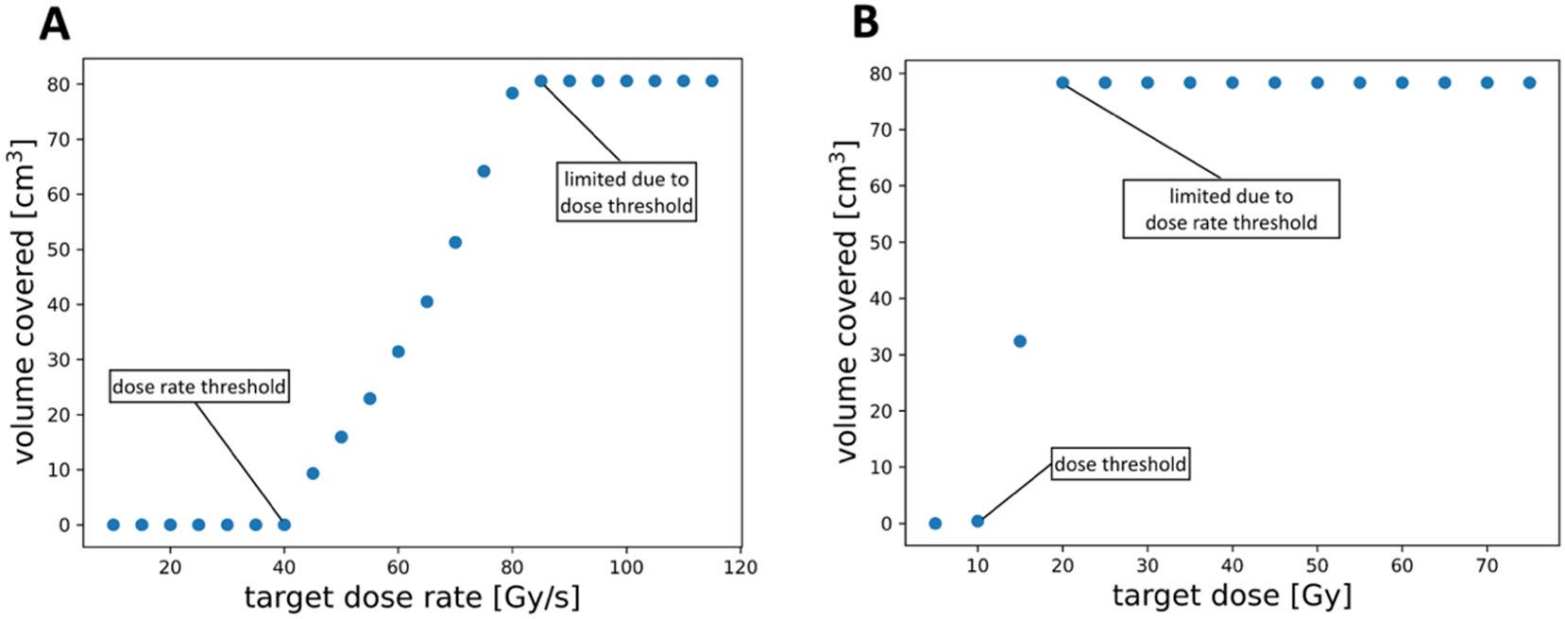
Volume covered by the FLASH effect. (**A**) As a function of target dose-rate for a target dose of 20 Gy. (**B**) As a function of target dose for a target dose-rate of 80 Gy/s.

Figure 4 demonstrates the total volume covered by the FLASH effect for protons at a target dose of 20 Gy as a function of target dose-rate and the total volume covered by the FLASH effect for protons at a target dose-rate of 80 Gy/s as a function of target dose.

### Average dose outside of target and dose volume histograms

**Fig. 5:**
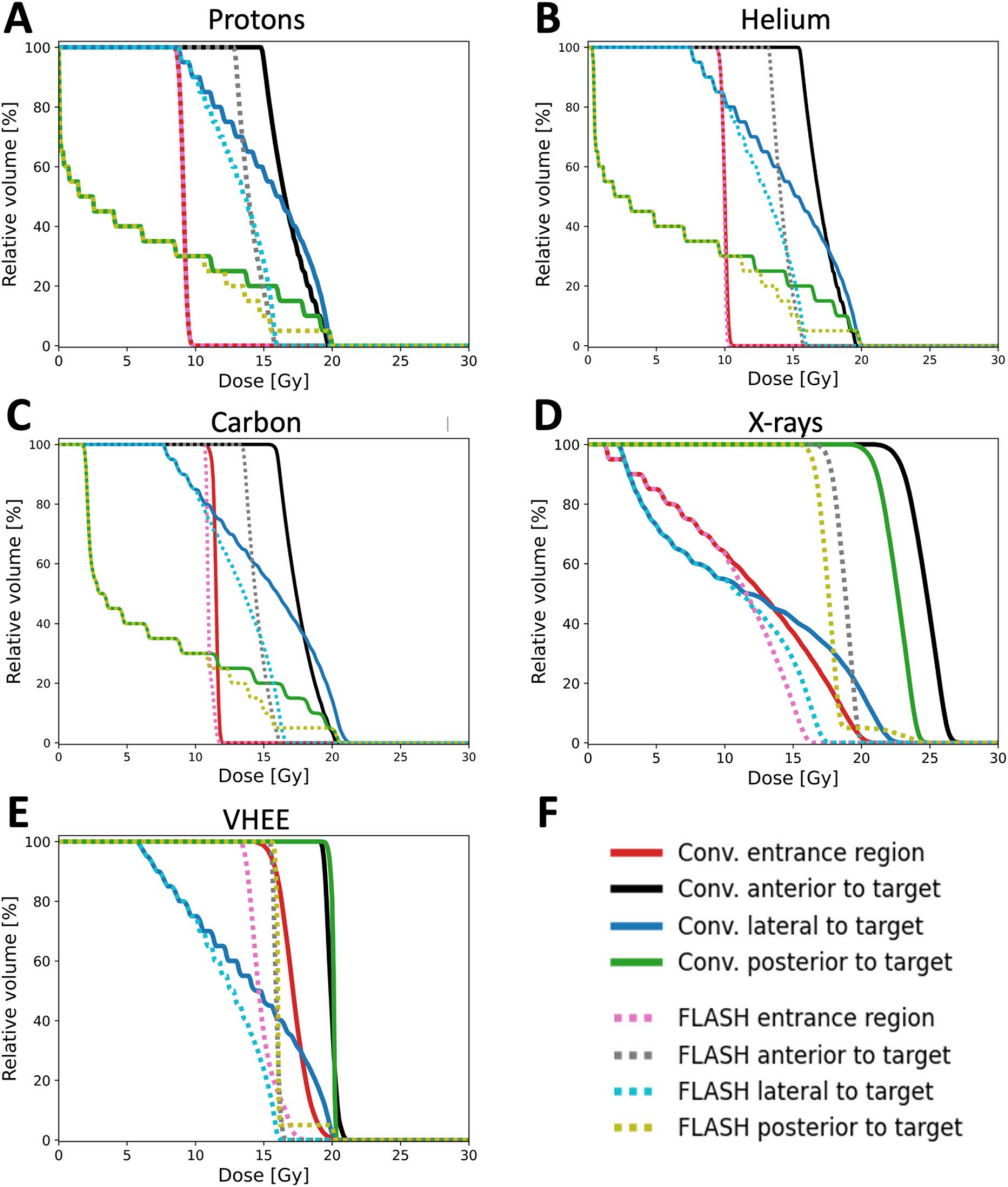
Dose-volume histograms for different regions of interest at target dose of 20 Gy. Comparison between conventional and FLASH dose-rate of 80 Gy/s. (**A**) Protons, (**B**) Helium, (**C**) Carbon, (**D**) X-rays, (**E**) VHEE, and (**F**) legend.

Table 2 presents the mean dose outside the target. The two columns show the values for a conventional dose-rate and for a FLASH dose-rate of 80 Gy/s, again for a target dose of 20 Gy. Figure 5 shows DVHs for different regions of interest, as defined in Fig. 1B, for all modalities, both for conventional dose-rates and FLASH dose-rates. Table 3 presents the ratio of the area of DVHs for FLASH dose-rate to the area of the DVHs for conventional dose-rate, for all modalities and regions of interest (ROIs).

**Table 2:** Average dose to healthy tissue for all modalities at conventional dose-rate and FLASH dose-rate of 80 Gy/s, with uncertainties propagated from the FLASH model parameters.

Modality	Average dose to healthy tissue [Gy]		Decrease [%]
	Conventional	FLASH	
Protons	3.1	2.9 (− 0.1)	6.5 (+ 3.2)
Helium	3.0	2.8 (± 0.1)	6.7 (+ 3.3 / − 3.4)
Carbon	3.0	2.9 (− 0.1)	3.3 (+ 3.3)
X-Rays	5.8	4.9 (± 0.2)	16 (+ 3 / − 4)
VHEE	6.9	6.1 (± 0.2)	12 (+ 2 / − 3)

**Table 3:** Area FLASH DVH divided by area conventional DVH for all modalities, with uncertainties propagated from the FLASH model parameters.

	Protons	Helium	Carbon	X-rays	VHEE
ROI 1	1.0	0.99 (+ 0.01 / − 0.02)	0.96 (+ 0.02 / − 0.03)	0.88 (± 0.03)	0.87 (+ 0.03 / − 0.04)
ROI 2	0.84 (+ 0.04 / − 0.05)	0.83 (± 0.04)	0.83 (+ 0.04 / − 0.05)	0.76 (+ 0.04 / − 0.05)	0.80 (+ 0.04 / − 0.05)
ROI 3	0.86 (+ 0.03 / − 0.05)	0.86 (± 0.04)	0.85 (± 0.04)	0.86 (+ 0.03 / − 0.04)	0.87 (+ 0.03 / − 0.04)
ROI 4	0.91 (+ 0.02 / − 0.03)	0.91 (+ 0.02 / − 0.03)	0.92 (+ 0.02 / − 0.03)	0.79 (+ 0.04 / − 0.05)	0.81 (+ 0.04 / − 0.05)

## Discussion

The most notable difference between the treatment modalities is the longitudinal dose deposition. As anticipated based on the characteristic depth dose deposition of each modality, higher dose deposition can be observed anterior and posterior to the target for X-rays and VHEE compared to hadrons. Consequently, this results in higher *FMF*s along the beam direction for the hadrons, as evidenced by the *FMF* plots for the different modalities in Fig. 2. This implies that the healthy tissue sparing effect will be lower for hadron than for electron or photon beams.

A comparison of both the transverse and longitudinal dose profiles at conventional dose-rates with those at FLASH dose-rates reveals that the dose outside the tumour region is reduced at FLASH dose-rates, resulting in a sharper dose fall-off, as illustrated in Fig. 3 for proton beams. In this case, the reduction extends up to approximately 1 cm laterally outside the target, 6 cm anterior to the target, and a couple of mm posterior to the target, where the threshold dose and threshold dose-rates are no longer met. The assumption that there is no tissue sparing at all in tumour tissue results in a jump in dose at the boundary between normal tissue and tumour tissue at FLASH dose-rates.

As anticipated, at conventional dose-rates, the hadrons exhibit a lower average dose compared to X-rays and VHEE. This is further substantiated by Fig. 2, which demonstrates that hadrons deposit less dose outside of the target. At FLASH dose-rate the average dose outside the target is observed to decrease for all modalities. Nevertheless, while X-rays and VHEE demonstrate a higher absolute and percentage decrease compared to hadrons, the average dose outside of the target for hadrons remains lower, even when comparing the conventional dose-rate for hadrons to the FLASH dose-rate for X-rays and VHEE and when accounting for the associated uncertainties of the FLASH model.

Although DVHs are typically used for the analysis of clinical dose distributions and the distributions in this study are not typical clinical ones, they give a clear visualisation of the differences between different ROIs and modalities, which is the reason that this analysis method is used. For the hadrons at conventional dose-rates ROIs 2 and 3, located anterior and lateral to the target, receive the highest dose out of the four ROIs. For X-rays and VHEE, these are ROIs 2 and 4, located anterior and posterior to the target. This is demonstrated by the DVHs in Fig. 5. The discrepancy can be attributed to the pronounced distal falloff resulting from the (spread-out) Bragg peak of the hadrons, which leads to a comparatively low dose deposition in ROI 4 relative to the other ROIs. When focusing on ROI 1, which is situated in the entrance region, it becomes evident that the dose is lowest in this region for X-rays, due to the build-up effect. The dashed lines in the figures illustrate the DVHs at FLASH dose-rate, where the difference between the solid lines and the dashed lines is proportional to the amount of healthy tissue sparing by the FLASH effect. Table 3 quantifies the difference between the DVHs at conventional and FLASH dose-rate, and the values indicate an average FMF inside the corresponding ROIs for the different modalities. With regard to hadrons, there is minimal to no healthy-tissue sparing in ROI1 at this target dose and dose-rate, in contrast to X-rays and VHEE. A difference is also observed in ROI4, where X-rays and VHEE show more healthy tissue sparing than the hadron modalities, due to the low dose distal to the target for hadrons as noted previously. In ROI2 and ROI3, the differences between modalities are less pronounced, and the uncertainty ranges overlap substantially because of the parameter uncertainties in the FLASH model.

For the hadrons, the minimal target dose and target dose-rate to achieve a sparing effect in the healthy tissue correspond to the *D*_*T*_ of mammalian without skin data set as provided earlier and *DR*_*T*_ of 40 Gy/s, with a slightly smaller dose-rate needed for carbon. For X-rays and VHEE, these values are comparable or slightly lower due to the difference in longitudinal dose deposition compared to hadrons. A more pronounced difference is observed for ROI1, where on average for hadrons a minimal target dose of approximately 20 Gy and a minimal target dose-rate of about 75 Gy/s is required to achieve healthy tissue sparing, which is approximately twice that of X-rays and VHEE. The minimum values required to achieve tissue sparing in ROI1 vary slightly between the hadrons, as can be seen in Table 1, with lower values for heavier particles. A clear distinction is observed in the required dose-rates, and the difference in the required dose between protons and carbon ions persists even after accounting for the associated uncertainties. A comparison of the two extremes, protons and carbon, shows that the FLASH effect manifests at a lower z for carbon at the same target dose and target dose-rate (see Fig. 2). Furthermore, ROI1 receives a higher dose for carbon, as shown by the DHVs. These findings indicate that the entrance and plateau dose are higher for carbon. When comparing depth–dose curves for monoenergetic beams of the three modalities, one would normally expect the plateau dose to be lower for the heavier particles. In this study, however, this trend is influenced by the energy spread applied to the hadron beams. For comparability reasons, the relative energy spread was the same for the modalities, but this results in a reduced peak-to-plateau ratio for heavier ions. This explains the difference and will be explored in further work.

As mentioned before RBE was not considered in this study. Including it would impact the physical dose distributions. For achieving a flat RBE-weighted longitudinal dose distribution with carbon ions for example, the physical dose in the SOBP would need to decrease in depth. This would impact the ratios between the target dose and entrance dose up to the target as well, which in its turn would change the amount of healthy tissue sparing by the FLASH effect. In the case of helium and carbon ions, using an RBE-weighted SOBP would typically require a lower physical dose in the plateau region. Within the current FLASH model, this would reduce the number of entrance-region voxels that meet the FLASH-triggering threshold conditions, thereby altering the predicted amount of healthy-tissue sparing. Consequently, including RBE would modify the quantitative differences observed between the modalities. While the present work focuses on a consistent comparison based on physical dose, investigating the impact of RBE in future studies will be important, especially for clinical translation. In addition, because LET influences biological effectiveness and may also modulate the FLASH response, the absence of LET dependence in the present model represents another limitation. Incorporating LET-dependent effects, like RBE, could similarly change the quantitative comparison between modalities.

Furthermore, as mentioned in the Materials and Methods section, identical beam spot sizes were assumed for all hadron modalities. This assumption was made to focus on beam–tissue interactions rather than differences in beam production. If smaller spot sizes were used for helium and carbon ions, sharper penumbras and higher dose conformity would be expected, leading to lower dose to surrounding healthy tissue. At the same time, this would reduce the volume of healthy tissue meeting the dose-threshold conditions associated with the FLASH effect.

Apart from particle-specific effects, it is also important to understand how the FLASH effect depends on the target dose and dose-rate. Figure 4 illustrates this relationship. As the target dose and target dose-rate increase, the volume covered by the FLASH effect also increases, as shown in Fig. 4. This is because a larger fraction of the volume will meet the dose and dose-rate thresholds.

For varying dose-rate the tissue volume impacted by FLASH is zero until the dose-rate threshold is met. With the increase in the dose-rate, the tissue volume increases, reaching a certain point where it becomes constant, as illustrated in Fig. 4A. This implies that the volume of tissue affected by the FLASH effect is constrained by the dose threshold requirements. A comparable trend can be seen for varying the dose, where the volume of tissue affected by the FLASH effect is constrained by the dose-rate threshold requirements, as can be seen in Fig. 4B. These constraints by the threshold values show their influence on the predicted extent of the FLASH effect. Lower dose or dose-rate thresholds would increase the volume of healthy tissue fulfilling the FLASH conditions. Conversely, higher thresholds would reduce the FLASH-affected volume. A lower dose threshold would lead to tissue sparing even at lower target doses and would additionally expand the possibility of employing multiple beam angles or fractionation while still meeting FLASH conditions. These dependencies highlight that the quantitative outcome is inherently tied to the assumed FLASH-triggering thresholds.

Building on the above considerations regarding dose-rate thresholds, we further note that this study assumes a step-function dependence on dose-rate because no experimentally validated model currently describes a continuous FLASH response. We acknowledge that this simplification does not reflect the likely gradual onset of the FLASH effect and using a smoother, gradual dose-rate dependence instead of a binary threshold would reduce the discontinuities observed in Fig. 4 and would likely lower the minimum dose-rate requirements listed in Table 1.

Furthermore, we note that in this study we employed the time-averaged dose-rate definition. This is a modest and simplifying approach, where the dose-rate in each voxel is computed as the voxel dose divided by the total irradiation time. This approach was chosen because the dose and dose-rate values were set and scaled in post-processing rather than derived from explicit machine-timing parameters, and therefore no fixed numerical values for layer-switching or spot-switching times were assumed. The use of the time-averaged dose-rate is consistent with the FLASH model by Böhlen et al. ^14^, which is based on in vivo datasets showing FLASH effects across a wide range of temporal delivery structures, provided that the time-averaged dose-rate exceeded the reported threshold of 40 Gy/s. We acknowledge that alternative dose-rate definitions (e.g., instantaneous or percentile-based) could yield higher dose-rate estimates in some voxels^16^, potentially causing additional voxels to meet the dose-rate threshold and thus increasing the predicted volume of spared healthy tissue.

## Conclusion

The main difference between the modalities in this simple configuration is the longitudinal dose distribution and the effect that this has on the amount of healthy tissue spared and the minimum target dose and target dose-rates required to achieve healthy tissue sparing. In general, the FMFs are typically lower for X-rays and VHEE compared to hadrons at the same target dose and target dose-rate, indicating a stronger tissue sparing with the FLASH effect. Nevertheless, the mean dose to healthy tissue at the FLASH dose-rate will remain higher than for hadrons at the conventional dose-rate. As X-rays and VHEE require a lower target dose to trigger the FLASH effect, specifically in the entrance region, potentially allowing the use of fractionation or different treatment angles in combination with FLASH, thereby reducing the administered dose per fraction or angle to the healthy tissue. In the case of hadrons, this study has shown that this will be less probable, however the advantage of the higher dose conformality and a lower dose to the surrounding healthy tissue remain. This indicated that a trade-off can be considered between the possibility of fractionation and the dose conformality. Further investigation is required to gain a deeper understanding of the dose threshold and dose-rate threshold values for triggering the FLASH effect, as they have a significant impact on the effectiveness of the FLASH effect and are of particular importance when considering the use of VHEE and X-rays in preference to hadrons. The simple configuration in this study has already demonstrated the considerable distinctions between the various treatment modalities. Further studies, possibly with realistic and clinical dose distributions, could additionally examine the use of different treatment angles, feasibility of fractionation, inclusion of LET and RBE, the effect of different dose-rate definitions, and the implementation of a smoother, continuous dose-rate dependence instead of the binary threshold used here. Such investigations would help clarify the impact of these variables on the efficacy and healthy tissue sparing of different treatment modalities.

## Data Availability

All simulation input files, analysis scripts, and processed data necessary to reproduce the results of this study are deposited in a public repository and assigned the DOI https://doi.org/10.34894/W7F0PK. The repository contains all files required for rerun of the simulations, post-processing of the simulation output, and generation of all figures and tables. The files will be made publicly accessible upon publication.
